# Three-Dimensional Collision Avoidance Method for Robot-Assisted Minimally Invasive Surgery

**DOI:** 10.34133/cbsystems.0042

**Published:** 2023-08-30

**Authors:** Ling Li, Xiaojian Li, Bo Ouyang, Hangjie Mo, Hongliang Ren, Shanlin Yang

**Affiliations:** ^1^ School of Management, Hefei University of Technology, Hefei, China.; ^2^ Key Laboratory of Process Optimization and Intelligent Decision-Making (Ministry of Education), Hefei University of Technology, Hefei, China.; ^3^ Philosophy and Social Sciences Laboratory of Data Science and Smart Society Governance (Ministry of Education), Hefei University of Technology, Hefei, China.; ^4^ Department of Biomedical Engineering, Faculty of Engineering, National University of Singapore, Singapore, Singapore.; ^5^ Department of Electronic Engineering, Shun Hing Institute of Advanced Engineering, The Chinese University of Hong Kong, Hong Kong, China.; ^6^ National Engineering Laboratory for Big Data Distribution and Exchange Technologies, Shanghai, China.

## Abstract

In the robot-assisted minimally invasive surgery, if a collision occurs, the robot system program could be damaged, and normal tissues could be injured. To avoid collisions during surgery, a 3-dimensional collision avoidance method is proposed in this paper. The proposed method is predicated on the design of 3 strategic vectors: the collision-with-instrument-avoidance (CI) vector, the collision-with-tissues-avoidance (CT) vector, and the constrained-control (CC) vector. The CI vector demarcates 3 specific directions to forestall collision among the surgical instruments. The CT vector, on the other hand, comprises 2 components tailored to prevent inadvertent contact between the robot-controlled instrument and nontarget tissues. Meanwhile, the CC vector is introduced to guide the endpoint of the robot-controlled instrument toward the desired position, ensuring precision in its movements, in alignment with the surgical goals. Simulation results verify the proposed collision avoidance method for robot-assisted minimally invasive surgery. The code and data are available at https://github.com/cynerelee/collision-avoidance.

## Introduction

In contemporary medical practice, minimally invasive surgery (MIS) has gained considerable prominence due to its numerous benefits, which include reduced patient trauma, accelerated recovery time, and shorter hospital stays. It has been broadly utilized in various medical fields, including, but not limited to, general surgery, urology, extracerebral procedures, and extracardiac procedures [1–3]. The sweeping advances in communication technology, robotic technology, microelectronic technology, and computer intelligent control technology have provided an important impetus to the evolution and comprehensive development of MIS [4]. In recent years, surgical robots, characterized by their flexibility, stability, and remote operation capabilities, have found extensive application in this field [5]. As a successful combination of robotic technology and minimally invasive techniques, the minimally invasive surgical robotic system can further enhance the success rate of the MIS [6] Notably, well-known surgical robotic systems such as Da Vinci [7], DLR MIRO [8], and SPORT Surgical System [9] have demonstrated utility in various surgical procedures, including puncture, suturing, and endoscope holding. These systems extend the surgical ability and enhance the quality of operation.

**Fig. 1.**
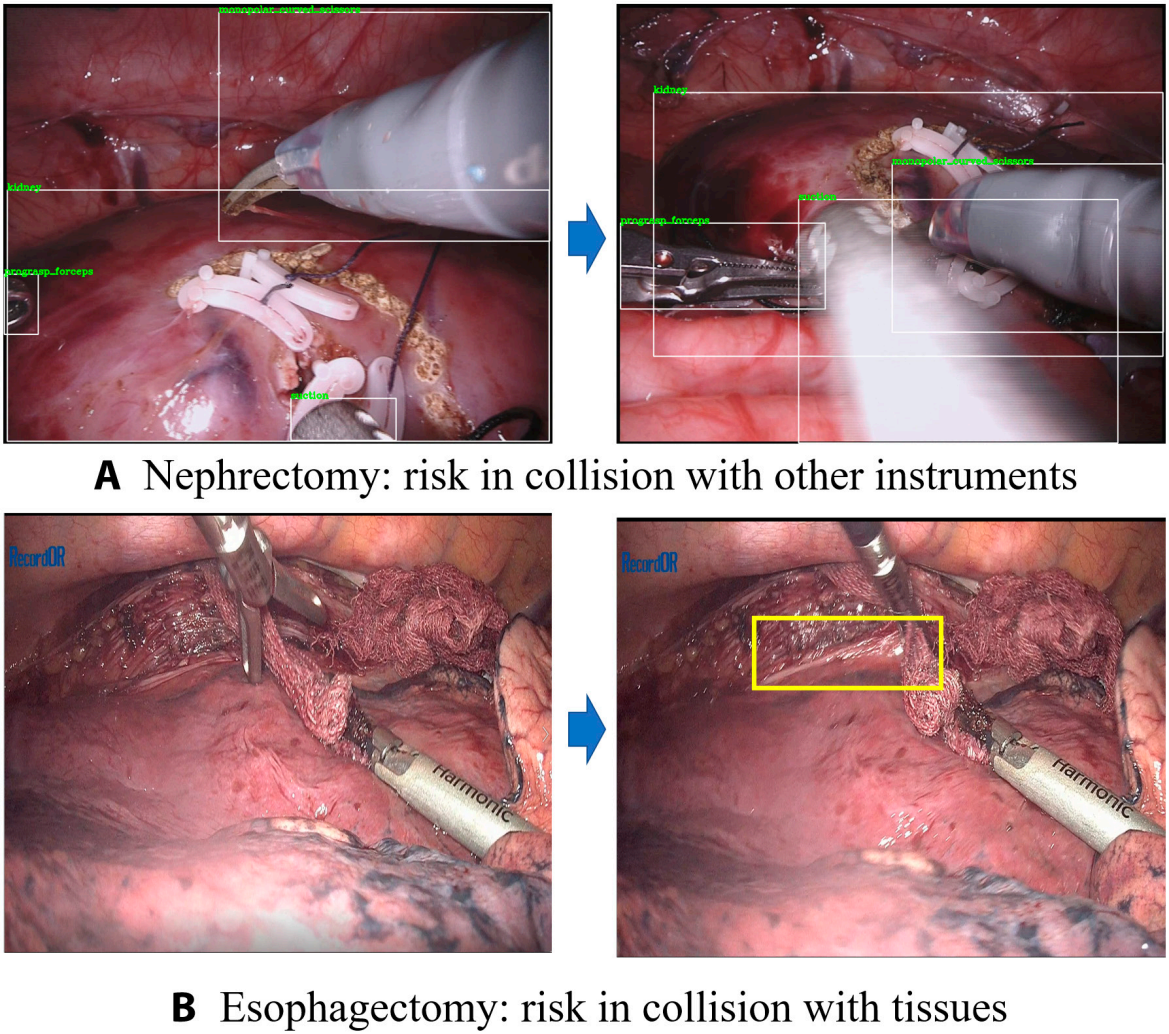
(A and B) Two kinds of collision risk.

Currently, surgical robot systems have undergone a significant transformation, evolving from nonautonomous entities to assistive tools, a development that is revolutionizing the landscape of surgical practice [10]. The scope of research and clinical application of such systems, particularly those that assist in holding endoscopes and manipulating tissues, is broadening. MIS typically necessitates the use of multiple surgical instruments and an endoscope. However, the surgeon’s capacity to simultaneously control these tools is limited, usually can only control 1 or 2 instruments. This limitation can be mitigated by leveraging surgical robotic systems to autonomously manage tasks such as holding endoscope or manipulating tissues [11–13]. Furthermore, robots are often more stable than medical staff when performing auxiliary operations [14]. This enhanced stability can alleviate the surgeon’s auxiliary workload, allowing them to focus on performing the primary surgical tasks more effectively. As shown in Fig. 1, there are primarily 2 types of collision risks: collision with surgeon-controlled instruments and collision with nontarget tissues. Figure 1A illustrates a scenario of nephrectomy, where there is a risk of collision between laparoscope (robot-controlled instrument) and the surgeon-controlled instrument. Figure 1B illustrates a scenario of esophagectomy, where there is a risk of collision between forceps (robot-controlled instrument) and tissues. There is a demand for a controller that can avoid active collisions with nongrasping tissues and surgeon-controlled instruments when the robot-controlled instrument is engaged in performing a task [15]. Thus, the development and refinement of these advanced controllers are of paramount importance in enhancing the safety and efficacy of robot-assisted MISs, paving the way for more precise and reliable surgical outcomes. A collision can cause damage to a robotic system program or harm the normal tissues in the body. It should be noted that the outside surgical scene is visible to a surgeon, so a collision between 2 or more surgical instruments outside the body can be avoided by the surgeon. However, the in vivo scene is not visible to a surgeon, and unskilled operations can cause collisions between surgical instruments in the body. Therefore, there has been a clinical requirement for a controller to avoid surgeon-controlled instruments and tissues when a robot-controlled instrument is idle, and to prevent active collision with nongrasping tissues and surgeon-controlled instruments when the robot-controlled instrument is performing a task.

The robotics field has seen numerous advancements in algorithms aimed at collision avoidance. The state-of-the-art methodologies in this arena encompass a variety of techniques, such as artificial potential fields [16], fuzzy logic control [17], ant colony algorithm [18], dynamic A^*^ search [19], Bug [20], and artificial neural networks [21]. These methods can be roughly divided into 2 categories. In the first category, obstacles’ poses and motions are required to be known before planning the path, which is difficult to achieve in the in vivo environment. In the second category, poses and motions of obstacles are provided as feedback in real time, and then the corresponding action is taken to avoid collision in real time. Because the behavior of a surgeon-controlled instrument is difficult to predict, the latter category is more suitable for surgical environments than the former one. Notable contributions to this field include the decentralized noncommunicating multi-agent collision avoidance algorithm based on deep reinforcement learning proposed by Chen et al. [22], and the collision avoidance vector-based control algorithm designed by Li et al. [23] to avert collisions with irregularly moving objects. Despite their innovations, these methods primarily address collision avoidance in a 2-dimensional (2D) dynamic environment.

Many 3D collision avoidance algorithms for robotic systems have been proposed recently. Alonso-Mora et al. [24] have introduced a method that addresses collision avoidance within a human–computer interaction environment. Franchi et al. [25] have proposed 3 unique control strategies for mitigating the 3D collision avoidance problem. There have also been significant developments reported in the realm of fully automated robotic systems, including unmanned aerial vehicles [26,27]. Most of these methods focus on static obstacles, and robots have a wide movement space. Surgical instruments or devices are inserted into the patient’s body through a small incision point known as the remote center of motion (RCM) of a surgical robot, thereby creating a significantly restricted range of movement. The RCM constraints and the limited surgical area impose restrictions on the range and velocity of surgical instrument’s motions. These limitations necessitate higher standards for in vivo collision avoidance methodologies. A review of related literature indicates that the issue of collision avoidance in robot-assisted MIS has been scarcely investigated. Notable efforts include Moccia et al.’s [15] design of forbidden region virtual fixtures to circumvent tool collision for da Vinci-like surgical robots, and Banach et al.’s [28] proposed active-constraint approach to prevent the clashing of surgical instruments. However, these studies primarily focus on surgical robotic systems operating under the master–slave control mode. They do not provide a collision avoidance strategy for the surgeon robot integration mode, nor do they consider the possibility of instrument collision with surrounding tissues. As such, these critical areas warrant further exploration and research.

To address the aforementioned limitations identified in extant literature, this paper introduces a novel 3D collision avoidance methodology specifically for robot-assisted MIS. This proposed method constitutes an enhanced version of the original collision avoidance vector-based control algorithm [23], drawing upon its dynamic characteristics while overcomzunique spatial constraints inherent in the surgical environment. Based on the kinematics, a collision-with-instrument-avoidance (CI) vector and a collision-with-tissues-avoidance (CT) vector are introduced to deal with the 3D collision avoidance problem in the in vivo environment. The CI vector ensures collision avoidance between the robot-controlled and surgeon-controlled instruments in 3 different directions with different parameters, while the CT vector ensures that the robot-controlled instrument will not collide with normal tissues. Additionally, a constrained-control (CC) vector is introduced to ensure that a robot-controlled instrument’s pose is close to the desired pose as much as possible in the in vivo environment. The results of the simulation in the MATLAB software environment show that the proposed method is effective in keeping a distance from a moving surgeon-controlled instrument and tissues with fixed incision point.

The main contributions of this paper can be summarized as follows:

1. A novel collision avoidance method framework, which can not only prevent a robot-controlled instrument and a surgeon-controlled instrument from colliding but also prevent a robot-controlled instrument from damaging tissues, is proposed.
2. The components of the CI vector can be used not only individually but also at the same time; different parameter combinations provide different collision avoidance effects.
3. A novel collision avoidance algorithm is developed under the proposed method framework, and users can set their own functions under the proposed method framework to achieve collision avoidance in robot-assisted MIS.

## Robot-Assisted Surgical System

The application of minimally invasive surgical robots can solve problems of crowded workstations, hand-held device shakes, and surgeon fatigue. In this paper, a novel robot-assisted surgical system to automatically prevent robot-controlled instrument collision with surgeon-controlled instrument and tissues is designed. First, the robotic system architecture is introduced in detail. Then, the kinematics with the RCM constraint is studied.

### System architecture

**Fig. 2.**
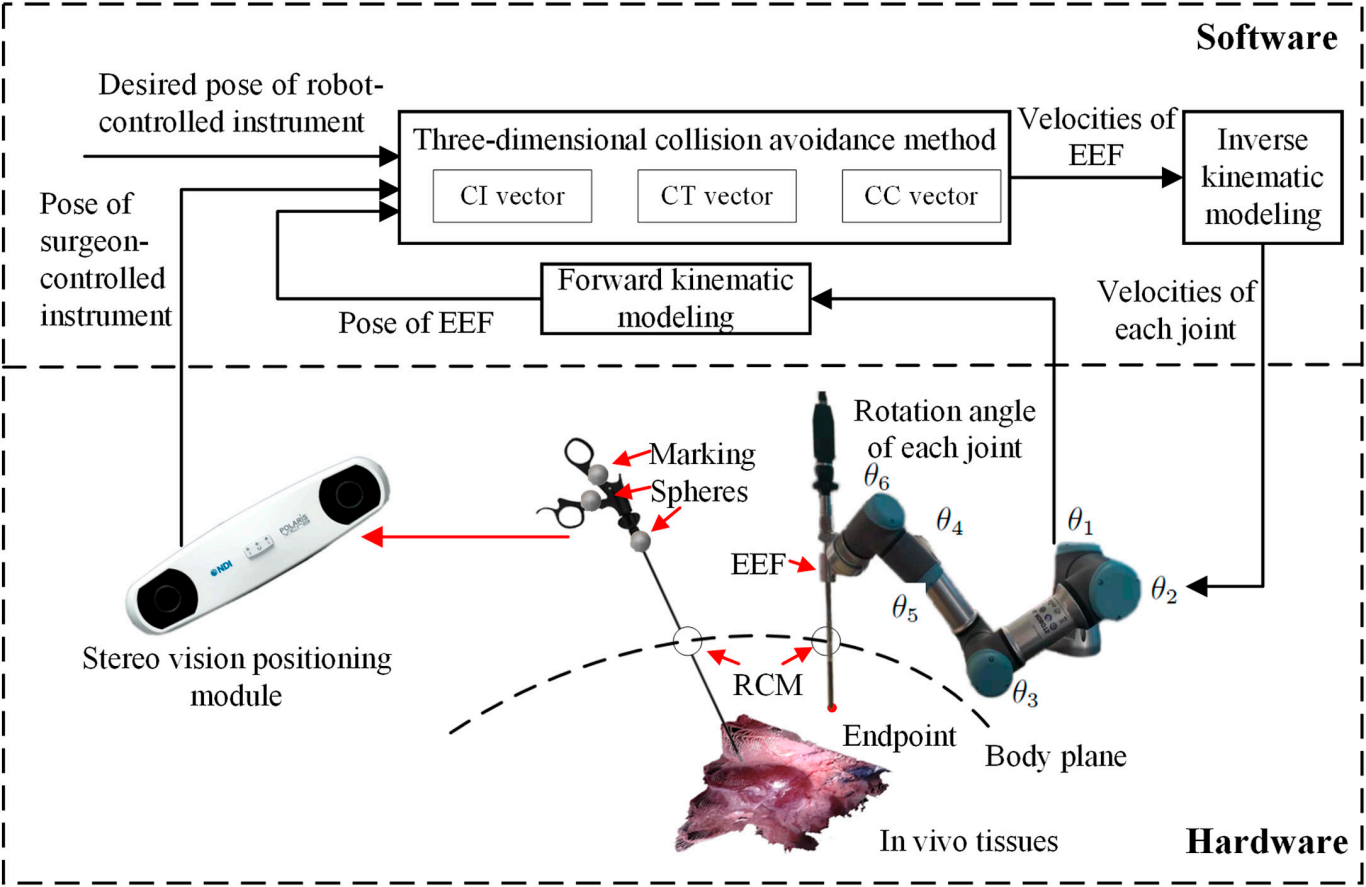
Robot-assisted surgical system architecture and its schematic.

The proposed robot-assisted surgical system is designed to avoid in vivo collision between robot-controlled instrument and surgeon-controlled instrument, and collisions between robot-controlled instrument and tissues. The schematic of the proposed system is shown in Fig. 2, where it can be seen that it consists of software and hardware parts. The hardware part consists of a minimally invasive surgical robot and a stereo vision positioning module. The minimally invasive surgical robot uses a 6-DOF (degree of freedom) collaborative robot named the UR5 manipulator [29]. It is formed by connecting 6 rotary joints in series to adjust the position of the end-effector (EEF) so that it can emulate a surgeon’s arm to operate surgical instruments and devices. The RCM represents a virtual joint of the UR5, which is at the incision point of the patient’s body plane. It constrains a robot-controlled instrument to move only around the incision point. A stereo vision positioning module is used to sense the surgeon-controlled instrument, and Polaris is used in the stereo vision positioning module [30].

**Fig. 3.**
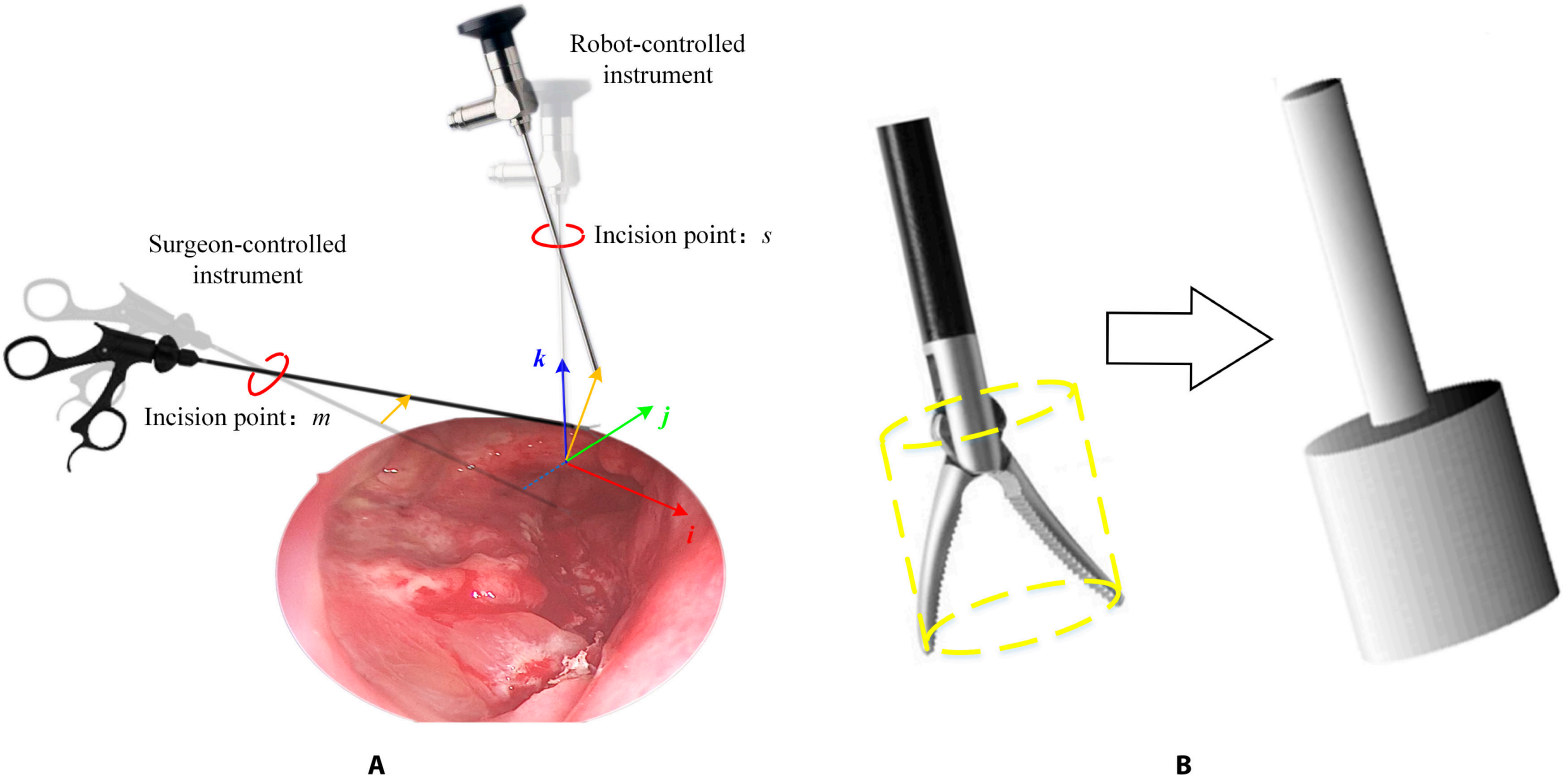
(A) Incision points with more detail. (B) Illustration of using 2 connected concentric cylinders to represent a surgical instrument.

The software part controls the robot’s movement in real time to prevent collisions inside the body. The desired position of the robot-controlled instrument’s endpoint and the real-time pose of the surgeon-controlled instrument denote the controller input. A 3D collision method is used to generate the velocity of the robot-controlled instrument’s endpoint, and the velocity of the robot’s each joint can be solved using inverse kinematics.

### Kinematic analysis with RCM constraint

The instrument controlled by the UR5 is inserted into the patient’s body through a small incision point during robot-assisted MIS. The RCM is a variable point on a given robot virtual link at the incision point. Therefore, operations on the patient’s body are limited by the incision point, which imposes a constraint on the motion of a robot, which is called the RCM constraint. Thus, to achieve automatic collision avoidance of a surgical robot, kinematics with RCM constraints should be analyzed first.

A set of the rotation angles of 6 joints of the UR5 is denoted as ***θ*** = [*θ*_1_ *θ*_2_ *θ*_3_ *θ*_4_ *θ*_5_ *θ*_6_]*^T^*, and the base coordinate system is established at the base of the UR5. The transformation matrix from link *i*-1 to line *i* is expressed as follows:

$$A_{i}^{i-1}\left(\theta_{i}\right)=\left[\begin{array}{cccc} \text{cos}\theta_{i} & -\text{cos}\alpha_{i}\text{sin}\theta_{i} & \text{sin}\alpha_{i}\text{sin}\theta_{i} & a_{i}\text{cos}\theta_{i}\\ \text{sin}\theta_{i} & \text{cos}\alpha_{i}\text{cos}\theta_{i} & -\text{sin}\alpha_{i}\text{cos}\theta_{i} & a_{i}\text{sin}\theta_{i}\\ 0 & \text{sin}\alpha_{i} & \text{cos}\theta_{i} & d_{i}\\ 0 & 0 & 0 & 1 \end{array}\right]$$ (1)

where *θ_i_*, *d_i_*, *α_i_*, and *a_i_* denote the Denavit–Hartenberg parameters of the UR5.

The coordinate transformation that describes the position and orientation of the EEF frame with respect to frame 6 is denoted as $T_{\mathrm{EEF}}^{6}$. Therefore, the position and orientation of EEF relative to the base frame is a function of ***θ***, which can be expressed as:

$$T_{EEF}^{0}=f\left(\theta \right)=\prod_{i=1}^{6}A_{i}^{i-1}\left(q_{i}\right)T_{EEF}^{6}=\left[\begin{array}{cccc} x_{EEF} & y_{EEF} & z_{EEF} & p_{EEF}\\ 0 & 0 & 0 & 1 \end{array}\right]$$ (2)

where ***x***_EEF_, ***y***_EEF_, and ***z***_EEF_ are the unit vectors of the 3 axes of the EEF relative to the base frame, and ***p***_EEF_ is the EEF position at the base coordinate system.

The coordinate transformation describing the position and orientation of the robot-controlled instrument’s endpoint with respect to the EEF frame is $T_{e}^{\mathrm{EEF}}$, so the position and orientation of the endpoint relative to the base frame are expressed as:

$$T_{e}^{0}=T_{EEF}^{0}T_{e}^{EEF}=\left[\begin{array}{llll} x_{e} & y_{e} & z_{e} & p_{e}\\ 0 & 0 & 0 & 1 \end{array}\right]$$ (3)

where ***x****_e_*, ***y****_e_*, and ***z****_e_* are the unit vectors of a frame attached to the robot-controlled instrument’s endpoint, and ***p****_e_* is the position vector of the robot-controlled instrument’s endpoint at frame 0.

Considering that the trocar’s position is fixed at the incision point, and the trocar can rotate only around the incision point, so the position of RCM ***p***_RCM_ is a constraint, and it holds that:

$$p_{EEF}=L\frac{p_{RCM}-p_{e}}{∥p_{RCM}-p_{e}∥}+p_{e}$$ (4)

$$z_{EEF}=\frac{p_{RCM}-p_{e}}{∥p_{RCM}-p_{e}∥}$$ (5)

where *L* denotes the length of the robot-controlled instrument.

Hand–eye coordination is critical to surgeons in a MIS. The orientation of a robot-controlled instrument should remain relatively fixed during a MIS. To ensure the stability of the orientation of surgical instruments controlled by a robot, ***x***_EEF_ and ***y***_EEF_ are defined as follows:

$$x_{EEF}=\left\{\begin{array}{ll} \frac{y_{0}\times z_{EEF}}{∥y_{0}\times z_{EEF}∥} & ,y_{0}\times z_{EEF}\neq 0\\ z_{0}z_{EEF}x_{EEF} & ,y_{0}\times z_{EEF}=0 \end{array}\right.$$ (6)

$$y_{EEF}=z_{EEF}\times x_{EEF}$$ (7)

where ***y***_0_ and ***z***_0_ are the unit vectors of the *y* axis and *z* axis at the base coordinate system.

By substituting Eqs. 4 to 7 into Eq. 3, $T_{EEF}^{0}$ can be denoted as a function of ***p****_e_*, and hence, there is $T_{EEF}^{0}=g\left(p_{e}\right)$. The inverse kinematics of a robot is expressed as:

$$\theta =f^{-1}\left(g\left(p_{e}\right)\right)$$ (8)

Differentiating Eq. 8 with respect to time, the relationship between the velocities of the endpoint and each joint can be obtained as:

$$\dot{\theta }=\frac{\partial f^{-1}\left(g\left(p_{e}\right)\right)}{\partial g\left(p_{e}\right)}\frac{\partial g\left(p_{e}\right)}{\partial p_{e}}{\dot{p}}_{e}$$ (9)

where $\dot{\theta }$ represents the velocity of each joint, and ${\dot{p}}_{e}$ represents the velocity of the robot-controlled instrument’s endpoint. According to Eq. 9, by setting the velocity of each joint, the desired velocity of robot-controlled instrument’s endpoint can be obtained.

## Collision Avoidance Method

In this section, a 3D collision avoidance vector method intended for robot-assisted MIS for preventing a robot-controlled instrument from colliding in the patient’s body is described. The 3D collision avoidance vector method consists of CI vector, CT vector, and CC vector. The CI and CT vectors aim at avoiding collisions of a robot-controlled instrument in vivo. The CI vector is used to avoid collisions between the robot-controlled instrument and surgeon-controlled instrument. The CT vector is designed to automatically control the velocity at the robot-controlled instrument’s endpoint to avoid collisions with the normal tissues. The CC vector can realize the maximum satisfaction of that robot-controlled instrument locating at desired pose. It should be noted that the desired pose is set artificially or by using other algorithms, and that is out of the scope of this paper.

Figure 3A shows a MIS scene where an endoscope is controlled by the EEF of a robot while a surgical instrument is operated by a surgeon. In Fig. 3, *m* and *s* indicate 2 incision points. The surgeon controls the surgical instrument into the patient’s body through *m*, while the robot controls an endoscope through *s*. Since the stereo vision positioning module can detect the position of marking spheres outside the body, with a known geometry of a surgical instrument, it is easy to infer the surgeon-controlled instrument’s pose in the patient’s body. As for a robot-controlled instrument, its pose can be obtained through the forward kinematics of the robot. To simplify the collision avoidance problem, the shaft of the surgical instrument is represented by a cylinder, and a circumscribed cylinder of the instrument’s tip represents the tip of the instrument, including the wrist and claspers. In this way, 2 connected concentric cylinders can be used to represent a surgical instrument, as shown in Fig. 3B.

The shortest distance between the robot-controlled instrument and surgeon-controlled instrument can be calculated as follows:

$$\begin{array}{ccc} d & = & \text{min}∥s_{j}-m_{j}∥\\ \left(s_{j}^{*},m_{j}^{*}\right) & = & \arg\text{min}∥s_{j}-m_{j}∥ \end{array}$$ (10)

where ***s****_j_* and ***m****_j_* are the points located on the surfaces of the robot-controlled instrument and surgeon-controlled instrument, respectively.

The CI vector is defined as follows:

**Definition 1** *(CI vector)*: The CI vector is introduced to avoid a collision between 2 surgical instruments, and it is defined as $\mathrm{CI}≜\sum_{i=1}^{3}‍CI_{i}$. Vector ***CI****_i_* follows the following rules:

(1) ***CI***_1_ ≜ *f*_1_(*d*)***i***, *f*_1_(*d*) ≥ 0.

(2) ***CI***_2_ ≜ *f*_2_(*d*)***j***, *f*_2_(*d*) ≥ 0.

(3) ***CI***_3_ ≜ *f*_3_(*d*)***k***, *f*_3_(*d*) ≥ 0.

(4) If there exists a constant *D*, such that *d* < *D*, then ${\dot{f}}_{1}\left(d\right)$, ${\dot{f}}_{2}\left(d\right)$, ${\dot{f}}_{3}\left(d\right)<0$; otherwise, ${\dot{f}}_{1}\left(d\right)={\dot{f}}_{2}\left(d\right)={\dot{f}}_{3}\left(d\right)=0$.

Constant *D* denotes the maximum distance needed to avoid the collision. The smaller the value of *D* is, the smaller the working range of the CI vector will be. The value of *D* can be adjusted according to the clinical requirements. Further, ***i***, ***j***, ***k*** are the unit vectors in the coordinate system at the frame of the robot-controlled instrument’s endpoint, and they are defined as:

$$\begin{array}{lll} i & = & \left\{\begin{array}{cc} \frac{s^{m}+z_{e}}{∥s^{m}+z_{e}∥} & ,\left(s^{m}+z_{e}\right)\cdot s_{e}>0\\ -\frac{s^{m}+z_{e}}{∥s^{m}+z_{e}∥} & ,\left(s^{m}+z_{e}\right)\cdot s_{e}<0 \end{array}\right.\\ j & = & z_{e}\times \left(\frac{s^{m}+z_{e}}{∥s^{m}+z_{e}∥}\times z_{e}\right)\\ k & = & -z_{e} \end{array}$$ (11)

where ***s****_e_* is the unit vector down the shaft of the surgeon-controlled instrument, and ***s****^m^* is the unit vector from $m_{j}^{*}$ to $s_{j}^{*}$, and it is calculated by:

$$s^{m}=\frac{s_{j}^{*}-m_{j}^{*}}{∥s_{j}^{*}-m_{j}^{*}∥}$$ (12)

**Fig. 4.**
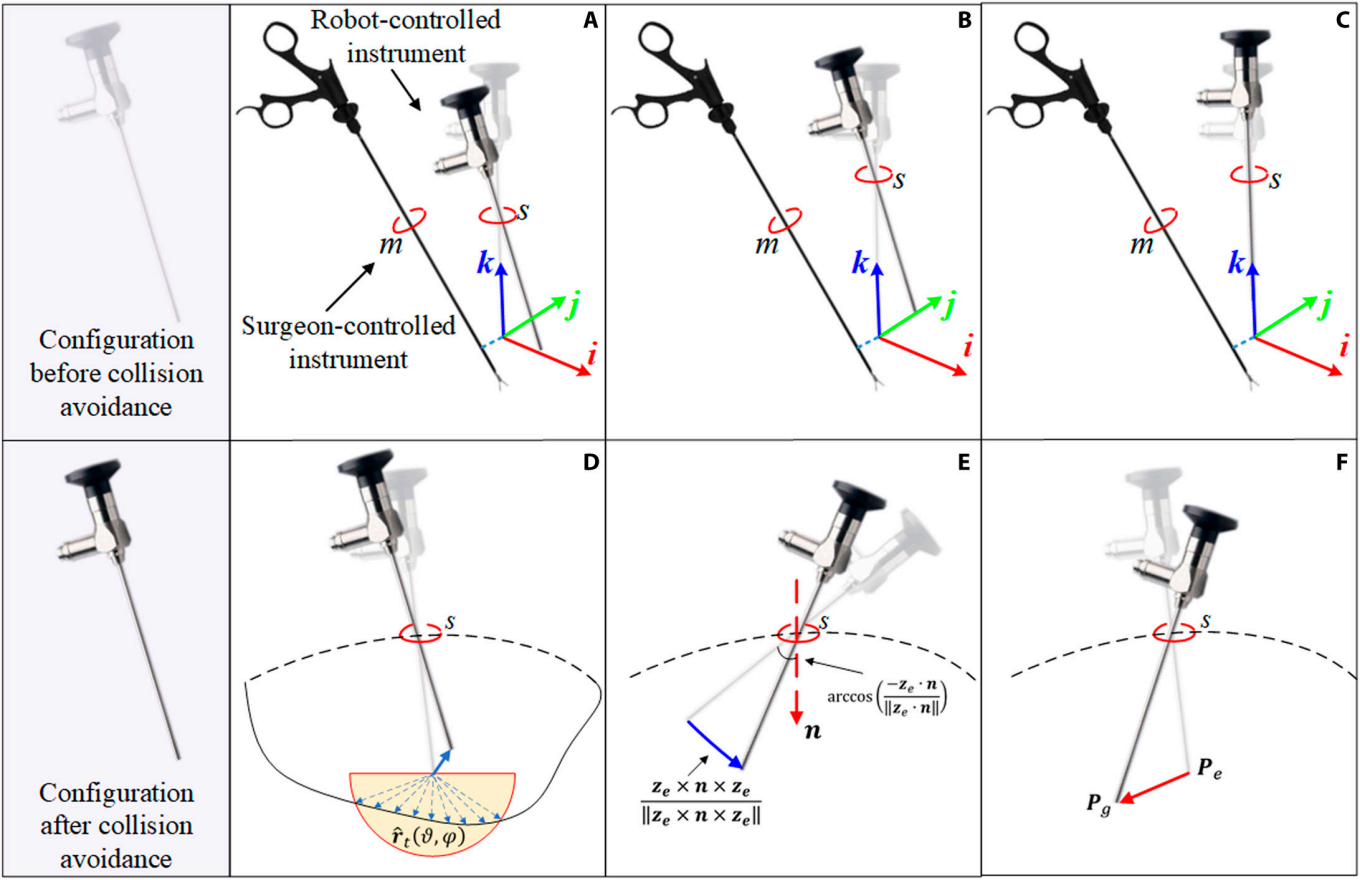
Illustration of *CI*_1_, *CI*_2_, *CI*_3_, *CT*_1_, *CT*_2_, and *CC* with their individual effects. (A) Effect of *CI*_1_. (B) Effect of *CI*_2_. (C) Effect of *CI*_3_. (D) Effect of *CT*_1_. (E) Effect of *CT*_2_. (F) Effect of *CC*.

**Remark 1**: ***CI***_1_ is introduced to drive the robot-controlled instrument to rotate around the surgeon-controlled instrument, thereby avoiding collisions with surgeon-controlled instrument. ***CI***_2_ is proposed to drive the robot-controlled instrument move away from the surgeon-controlled instrument to avoid collision. ***CI***_3_ is defined to lift the robot-controlled instrument along the *z* axis to avoid a collision. The illustration of ***CI***_1_, ***CI***_2_, and ***CI***_3_ with their individual effects is shown in Fig. 4A to C.

Suppose that the 3D shape of tissues in vivo is denoted as *r_t_*(*ϑ*, *φ*), and the spherical coordinate system is set at the endpoint of the robot-controlled instrument (i.e., endoscope in this section). *r_t_*(*ϑ*, *φ*) can be obtained by a 3D endoscope [31] or a depth perception method, such as improved simultaneous localization and mapping [32], improved structure from motion [33], and deep learning-based depth estimation [34].

Further, the CT vector is introduced to prevent collisions between a robot-controlled instrument and normal tissues.**Definition 2** (*CT vector*): The proposed CT vector is defined as $\mathrm{CT}≜CT_{1}+CT_{2}$, where

$${\mathrm{CT}}_{1}≜\left\{\begin{array}{cc} \frac{\sum_{0}^{\mathrm{num}}‍f_{4}\left(r_{t}\left(\vartheta ,\varphi \right)\right){\overset{ˆ}{r}}_{t}\left(\vartheta ,\varphi \right)}{\mathrm{num}} & ,\mathrm{num}>0\\ 0 & ,\mathrm{num}=0 \end{array}\right.$$ (13)

$${\mathrm{CT}}_{2}≜f_{5}\left(z_{e},n\right)\frac{z_{e}\times n\times z_{e}}{∥z_{e}\times n\times z_{e}∥}$$ (14)

where ${\hat{r}}_{t}(\vartheta ,\varphi ))$ denotes the unit vector from the surgeon-controlled instrument’s endpoint to the point (*r_t_*(*ϑ*, *φ*), *ϑ*, *φ*); num is the number of cloud points that meet condition of *r_t_*(*ϑ*, *φ*) ≥ *D*_1_; it holds that *f*_4_(*r_t_*(*ϑ*, *φ*)) < 0. ***n*** is the unit direction of the normal vector of the body plane through *s*. If $\arccos\left(\frac{-z_{e}\cdot n}{∥z_{e}\cdot n∥}\right)>\beta$, then *f*_5_(***z****_e_*, ***n***) > 0; otherwise, *f*_5_(***z****_e_*, ***n***) = 0. Additionally, as the value of $\arccos\left(\frac{-z_{e}\cdot n}{∥z_{e}\cdot n∥}\right)$ increases, *f*_5_(***z****_e_*, ***n***) decreases. It should be noted that both *D*_1_ and *β* are thresholds set according to the actual situation requirements.

**Remark 2**: ***CT***_1_ is proposed to avoid a robot-controlled instrument collision with the normal tissues. The tissues close to the robot-controlled instrument have the repulsion force to the robot-controlled instrument, and the integral of the repulsion force represents the velocity of the surgical instrument’s movement needed to avoid collisions. ***CT***_2_ is defined for keeping the robot-controlled instrument inside the patient’s body. The illustration of the exact functions with their individual effect is shown in Fig. 4D and E.

In a robot-assisted MIS, a robot-controlled instrument usually needs to perform simple tasks, such as holding an endoscope and grasping. In this case, a robot-controlled instrument has a target pose, which is task-driven, and usually set by a surgeon or obtained by other algorithms. Assuming that ***p****_g_* is the desired position of a robot-controlled instrument, to maintain the target pose, a CC vector is defined as follow:

**Definition 3** (*CC vector*): The proposed CC vector is expressed as:

$$\mathrm{CC}≜f_{6}\left(p_{g},p_{e}\right)\frac{p_{g}-p_{e}}{∥p_{g}-p_{e}∥}$$ (15)

where *f*_6_(***p****_g_*, ***p****_e_*) ≥ 0. As the distance between ***p****_g_* and ***p****_e_* decreases, the value of *f*_6_(***p****_g_*, ***p****_e_*) also decreases. Due to the CC vector, the robot-controlled instrument can get back to the target position after avoiding collisions. Figure 4F shows the effect of the CC vector.

Finally, the velocity of the robot-controlled instrument’s endpoint ${\dot{p}}_{e}$ is obtained by the proposed collision avoidance method as follows:

$${\dot{p}}_{e}≜\xi \left(\xi \left(\mathrm{CI}\right)+\xi \left(\mathrm{CT}\right)+\xi \left(\mathrm{CC}\right)\right)$$ (16)

In addition, function *ξ*(***x***) is defined as:

$$\xi \left(x\right)=\left\{\begin{array}{ll} x & ,∥x∥\leq V_{\max}\\ \frac{V_{\max}}{∥x∥}x & ,∥x∥>V_{\max} \end{array}\right.$$ (17)

where *V*_max_ represents a constant. By substituting Eq. 16 into Eq. 9, the velocity of robot’s each joint can be obtained.

In practical applications, different functions of CI, CT, and CC vectors can be defined to meet the actual clinical requirements.

## Collision Avoidance Algorithm

In this section, a group of functions that can be used for clinical collision avoidance is presented. These functions are based on the framework of the proposed collision avoidance method.

When the condition of *d* < *D* is satisfied, in the CI vector, functions *f*_1_(*d*), *f*_2_(*d*) and *f*_3_(*d*) are defined as follows:

$$\left\{\begin{array}{lll} f_{1}\left(d\right) & = & \frac{K_{1}\left(D-d\right)D^{2}}{d^{4}}\\ f_{2}\left(d\right) & = & \frac{K_{2}\left(D-d\right)D^{2}}{d^{4}}\\ f_{3}\left(d\right) & = & \frac{K_{3}\left(D-d\right)D^{2}}{d^{4}}e^{\frac{4∥s_{j}^{*}-P_{e}∥}{∥s-p_{e}∥}} \end{array}\right.$$ (18)

where *K*_1_, *K*_2_, and *K*_3_ denote positive constants; when *d* ≥ *D*, it holds that all the functions are equal to zero, i.e., *f*_1_(*d*) = *f*_2_(*d*) = *f*_3_(*d*) = 0. The collision avoidance effect varies when parameters *K*_1_, *K*_2_, and *K*_3_ change. Users can set different parameters to adjust the strategies and directions of the CI vector.

In the CT vector, functions *f*_4_(*r_t_*(*ϑ*, *φ*)) and *f*_5_(***z****_e_*, ***n***) are designed under the proposed framework as follows:

$$f_{4}\left(r_{t}\left(\vartheta ,\varphi \right)\right)=\left\{\begin{array}{cc} -K_{4}\cos\left(\frac{\pi \left(r_{t}\left(\vartheta ,\varphi \right)\right)}{2D_{1}}\right) & ,r_{t}\left(\vartheta ,\varphi \right)<D_{1}\\ 0 & ,r_{t}\left(\vartheta ,\varphi \right)\geq D_{1} \end{array}\right.$$ (19)

$$f_{5}\left(z_{e},n\right)=\left\{\begin{array}{cc} K_{5}\text{In}\left(\frac{-z_{e}\cdot n}{∥z_{e}\cdot n∥}\right) & ,\text{arccos}\left(\frac{-z_{e}\cdot n}{∥z_{e}\cdot n∥}\right)>\beta \\ 0 & ,\text{arccos}\left(\frac{-z_{e}\cdot n}{∥z_{e}\cdot n∥}\right)\leq \beta \end{array}\right.$$ (20)

where *K*_4_ and *K*_5_ denote positive constants.

For the CC vector, function *f*_6_(***p****_g_*, ***p****_e_*) is defined to keep the robot-controlled instrument at the target position, and it is expressed as:

$$f_{6}\left(p_{g},p_{e}\right)=K_{6}∥p_{g}-p_{e}∥$$ (21)

where *K*_6_ denotes a positive constant that limits the size of the CC vector.

The pseudo-code of the proposed collision avoidance algorithm is given in Algorithm 1.


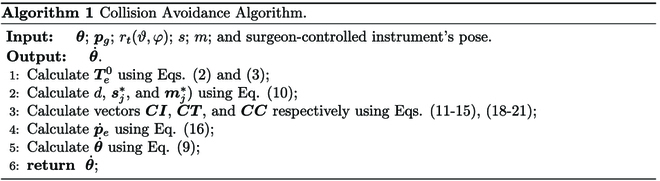


## Experiments

### Experimental methods

**Fig. 5.**
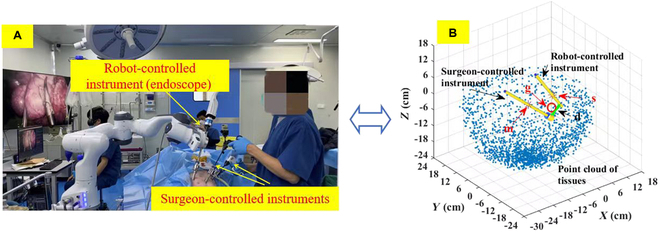
(A and B) Simulation setup.

To validate the proposed collision avoidance method, simulations were performed in the MATLAB software environment. All simulated objects were in the same global coordinate system. The simulation setup is displayed in Fig. 5. Figure 5A illustrates the scenario of the proposed algorithm being used in clinical practice. A laparoscope is controlled by robot, while instruments are controlled by the surgeon. The robot needs to adjust the laparoscope pose to keep the instruments within the field of view. Simultaneously, it should employ collision avoidance strategies to prevent the laparoscope from colliding with the surgeon-controlled instruments during the adjustment process. Figure 5B simulates the depicted scenario in Fig. 5A, where connected concentric cylinders represent the robot-controlled instrument and surgeon-controlled instrument, and ***m*** and ***s*** with a symbol ∗ denote the incision points. Both robot-controlled and surgeon-controlled instruments are constrained by the incision points. In Fig. 5B, the red circle denotes the desired position of the robot-controlled instrument’s endpoint; the green line represents the shortest distance between 2 instruments, and *d* stands for distance; the blue point clouds represent the tissues in the patient’s body. The relevant simulation data are shown in Table.

**Table.** Environment variable settings.

Environment variable	Value
Position of ***s***	***s*** = [0 0 0]*^T^* (cm)
Position of ***m***	***m*** = [−10 0 0]*^T^* (cm)
Position of ***p****_g_*	***p****_g_* = [0 0 − 5]*^T^* (cm)
Length of surgeon-controlled instrument’s shaft	28 cm
Length of robot-controlled instrument’s shaft	28 cm
Length of surgeon-controlled instrument’s tip	3 cm
Length of robot-controlled instrument’s tip	3 cm
Opening degree of surgeon-controlled instrument’s tip	Random positive number less than 60 degrees
Opening degree of robot-controlled instrument’s tip	0 degree
Radius of surgeon-controlled instrument’s shaft	0.5 cm
Radius of robot-controlled instrument’s shaft	0.5 cm
Initial position of robot-controlledinstrument’s endpoint ***p****_e_*	***p****_e_* = [1.38 1.02 − 10]*^T^* (cm)
Trajectory of surgeon-controlled instrument’s endpoint	$\left[\begin{array}{c} 3+10\text{sin}\left(\frac{t}{50}\pi +1.01\pi \right)\\ 4+10\text{cos}\left(\frac{t}{50}\pi +1.01\pi \right)\\ -10+\text{sin}\left(\frac{t}{50}\pi +1.01\pi \right) \end{array}\right]$
Point cloud of tissues	1952 points that randomly sampled in the hemisphere with ***m*** asthe center of the sphere and 15 cm as the radius
Sampling frequency	2 Hz
Sampling time	150 s

First, the effect of the CI vector with different components was analyzed. To show the impact of ***CI***_1_ on collision avoidance between surgical instruments, *K*_1_ was set to zero (i.e., the baseline), 0.1, 1, 3, and 4, in turn. The other parameters were set as follows: *K*_2_ = *K*_3_ = *K*_4_ = *K*_5_ = 0, and *K*_6_ = 0.1. In these cases, the effect of ***CI***_1_ in avoiding collisions between instruments was analyzed. Similarly, to demonstrate the impact of ***CI***_2_ on collision avoidance between surgical instruments, *K*_2_ was set to zero (i.e., the baseline), 0.1, 1, 5, 25, and 27, in turn. The other parameters were set as follows: *K*_1_ = *K*_3_ = *K*_4_ = *K*_5_ = 0, and *K*_6_ = 0.1; *K*_3_ was set to zero (i.e., the baseline), 0.1, 1, 5, 20, and 27, in turn. The parameter settings of *K*_1_ = *K*_2_ = *K*_4_ = *K*_5_ = 0 and *K*_6_ = 0.1 were used to analyze the effect of ***CI***_3_ on collision avoidance between surgical instruments. In the above simulations, *D* was set to 3 cm and *V*_max_ was set to 1 cm/s.

Next, the effect of the CT vector on avoiding collisions between a robot-controlled instrument and tissues was tested. Since ***CT***_1_ was mainly used to control robot-controlled instrument moving away from the tissues along the normal vector of the tissues, in the simulation of testing the performance of ***CT***_1_, only tissues were considered, without considering the surgeon-controlled instrument. The simulation parameters were set as follows: *K*_1_ = *K*_2_ = *K*_3_ = *K*_5_ = 0, *K*_6_ = 0.02, *D*1 = 2 cm, *V*_max_ = 1 cm/s, and *K*_4_ was set to zero (i.e., the baseline), 0.05, 0.1, 1, and 5, in turn. ***CT***_2_ should prevent a robot-controlled instrument from moving out of the body and colliding with the upper end of tissues during the movement of a robot-controlled instrument. In these simulations, parameters were set as follows: *K*_1_ = 1, *K*_2_ = *K*_3_ = *K*_4_ = 0, *K*_6_ = 0.001, *V*_max_ = 1 cm/s, *D* = 3 cm, $\beta =\frac{\pi }{4}$, *K*_5_ was set to zero (i.e., the baseline), 0.1, 1, 5, and 10, in turn.

Finally, an optimal parameter combination under the current environmental conditions was determined according to the analysis and simulation results. Under *K*_6_ = 0.1, *D* = 3 cm, *D*_1_ = 2 cm, and *V*_max_ = 1 cm/s, the other parameters were set as follows: *K*_1_ = 3, *K*_2_ = 5, *K*_3_ = 5, *K*_4_=5, *K*_5_ = 10.

### Results

#### Impact of CI vector on collision avoidance between robot-controlled and surgeon-controlled instruments

**Fig. 6.**
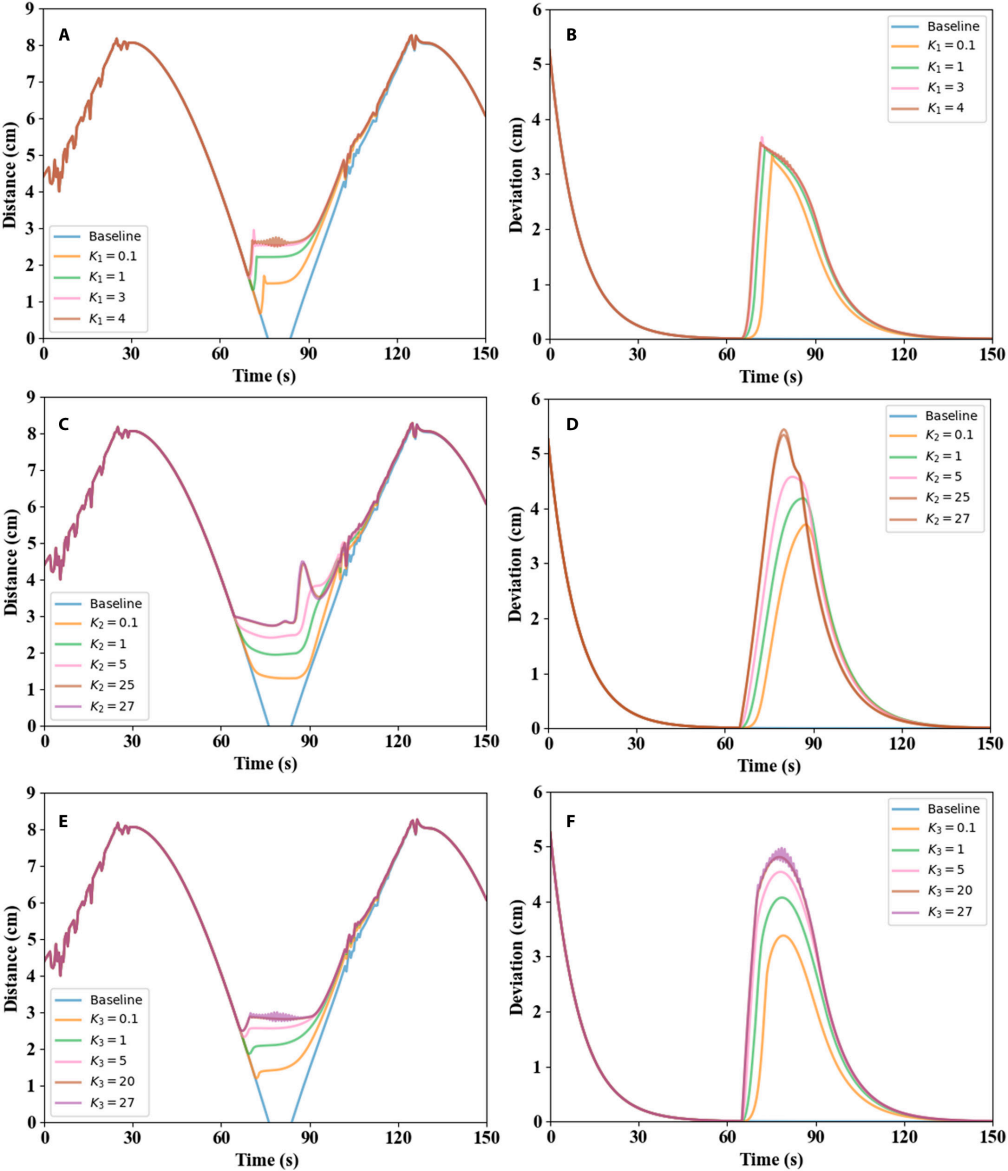
Performance comparison under different *K*_1_, *K*_2_, and *K*_3_. (A, C, and E) Shortest distance between 2 instruments. (B, D, and F) Deviation of the robot-controlled instrument’s endpoint from the desired position.

Vector CI prevented collisions between instruments in 3 mutually perpendicular directions. As for ***CI***_1_, over time, the shortest distance between 2 surgical instruments in the simulations was as shown in Fig. 6A. The baseline result showed that when a robot did not perform any collision avoidance operations, and a robot-controlled instrument moved directly to the target position, the 2 instruments would collide. Besides, Fig. 6A shows that within a certain range, the larger the value of *K*_1_ was, the greater the distance between the 2 instruments was. In addition, a large *K*_1_ could cause shaking of the robot-controlled instrument. As shown in Fig. 6A, the robot-controlled instrument jittered at *K*_1_ = 4, and the collision avoidance performance was similar when *K*_1_ = 3 and *K*_1_ = 4. The deviation of the robot-controlled instrument’s endpoint from the desired position ***p****_g_* is displayed in Fig. 6B, where it can be seen that when the distance between the 2 instruments was less than 3 cm, the robot-controlled instrument would leave the desired position for a short time, and when the danger would be avoided, the robot-controlled instrument would return to the desired position. The larger the value of *K*_1_ was, the greater the deviation was. The maximum deviation between *K*_1_ = 3 and *K*_1_ = 0.1 was approximately 0.5 cm, which was very small and had a little impact on the target task. Therefore, *K*_1_ = 3 was selected as a value that could provide the best collision avoidance effect in the analyzed situation.

The value of *K*_2_ affected the collision avoidance effect of ***CI***_2_. As shown in Fig. 6C and D, ***CI***_2_ ensured fast collision avoidance between the robot-controlled instrument and surgeon-controlled instrument. Within a certain range, i.e., when the value of *K*_2_ is less than 27 in the simulated environment, the greater the value of *K*_2_ was, the better the collision avoidance performance and the larger deviation from the robot-controlled instrument to the desired position were. As the value of *K*_2_ increased, the collision avoidance performance showed a convergence trend. As presented in Fig. 6C and D, the collision avoidance performance was almost the same under *K*_2_ = 25 and *K*_2_ = 27. As presented, a too-large value of *K*_2_ would cause a large deviation of the robot-controlled instrument’s endpoint from the desired position. When *K*_2_ = 5, the robot-controlled instrument could perform significantly in collision avoidance, and the minimum distance between robot-controlled and surgeon-controlled instruments was equal to *D*. Therefore, *K*_2_ = 5 was selected as an optimal value in the analyzed environment.

The value of *K*_3_ affected the performance of ***CI***_3_. Figure 6E and F shows how *K*_3_ affected the collision-avoiding performance between the 2 instruments. The same as for *K*_1_ and *K*_2_, when *K*_3_ < 27, the greater the value of *K*_3_ was, the better collision avoidance performance and the larger deviation from the robot-controlled instrument to the desired position were. In addition, a good collision avoidance effect and a small offset were achieved by setting *K*_3_ = 5.

**Fig. 7.**
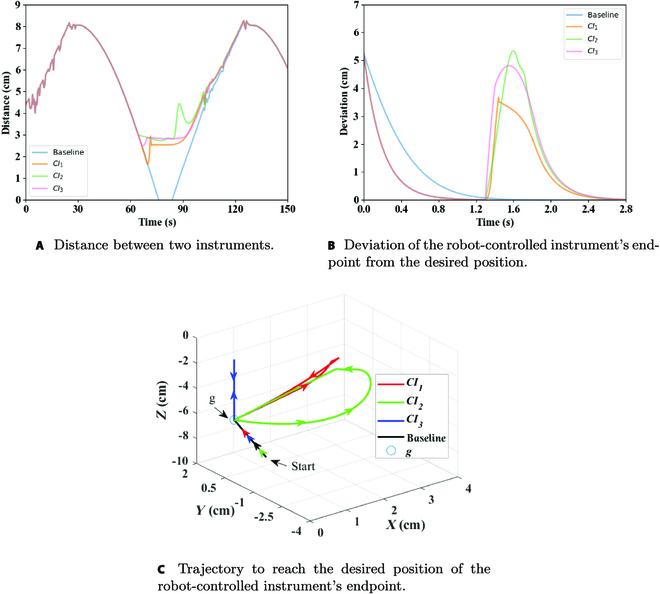
(A to C) Performance comparison of components *CI*_1_, *CI*_2_, and *CI*_3_.

The performance of ***CI***_1_, ***CI***_2_, and ***CI***_3_ was compared when they reached the farthest collision avoidance distance. In the analysis of ***CI***_1_, only ***CI***_1_ was used to achieve collision avoidance, and *K*_1_ = 3. In the analysis of ***CI***_2_, only ***CI***_2_ was used to achieve collision avoidance, and *K*_2_ = 25. Last, in the analysis of ***CI***_3_, only ***CI***_3_ was used to achieve collision avoidance, and *K*_3_ = 20. As shown in Fig. 7A and B, all ***CI***_1_, ***CI***_2_, and ***CI***_3_ could avoid collisions; ***CI***_2_ performed the best in collision avoidance, but it had the largest deviation of the robot-controlled instrument from the desired position. The reason why ***CI***_3_ took the least time among the CI vector was that its collision avoidance direction was closer to the direction of the desired position. Figure 7C shows the trajectory to reach the desired position of the robot-controlled instrument’s endpoint; it shows the actual effect of the 3 components of the CI vector.

#### Impact of CT vector on collision avoidance between robot-controlled instrument and tissues

**Fig. 8.**
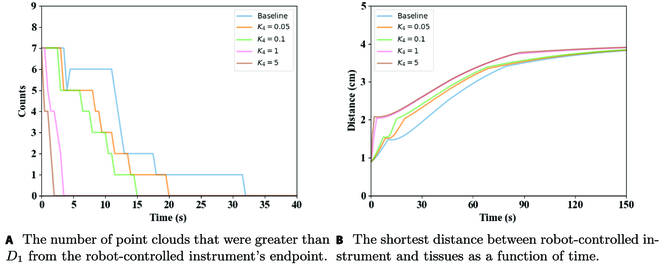
(A and B) Performance comparison under different *K*_4_.

To test the performance of ***CT***_1_, the number of point clouds that were greater than *D*_1_ from the robot-controlled instrument’s endpoint were analyzed, and the results are shown in Fig. 8A. As shown in Fig. 8A, when there was no CT vector, the robot-controlled instrument could not avoid collision with tissues when it moved to the desired position. The results showed that the number of point clouds of the baseline was always above the other results, and it took about 33 s to get away from the tissues. As the value of *K*_4_ increased, the time needed to avoid collisions between robot-controlled instrument and tissues shortened. Figure 8 shows the shortest distance between the robot-controlled instrument and tissues as a function of time. The value of *K*_4_ = 5 was chosen as the best ***CT***_1_ under the considered conditions.

**Fig. 9.**
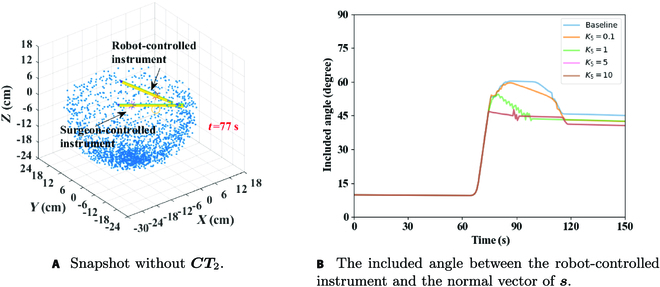
(A and B) Performance comparison under different *K*_5_.

Vector ***CT***_2_ was used to prevent a robot-controlled instrument from being too large under the normal vector of *s* in the process of avoiding surgeon-controlled instrument, causing the danger of collision with the upper part of tissues. Figure 9A shows a snapshot without *K*_5_, where it can be seen that the robot-controlled instrument had the risk of puncturing tissues. Thus, if the angle between the robot-controlled instrument and the normal vector could be decreased, the risk of puncturing tissues could be reduced. Figure 9B compares results of the included angle between robot-controlled instrument and the normal vector of ***s*** under different *K*_5_. The larger the value of *K*_5_ was, the smaller the angle and risk of collision with tissues would be. Therefore, *K*_5_ = 10 was selected as the optimal ***CT***_2_ under the analyzed condition.

#### Results of proposed algorithm

**Fig. 10.**
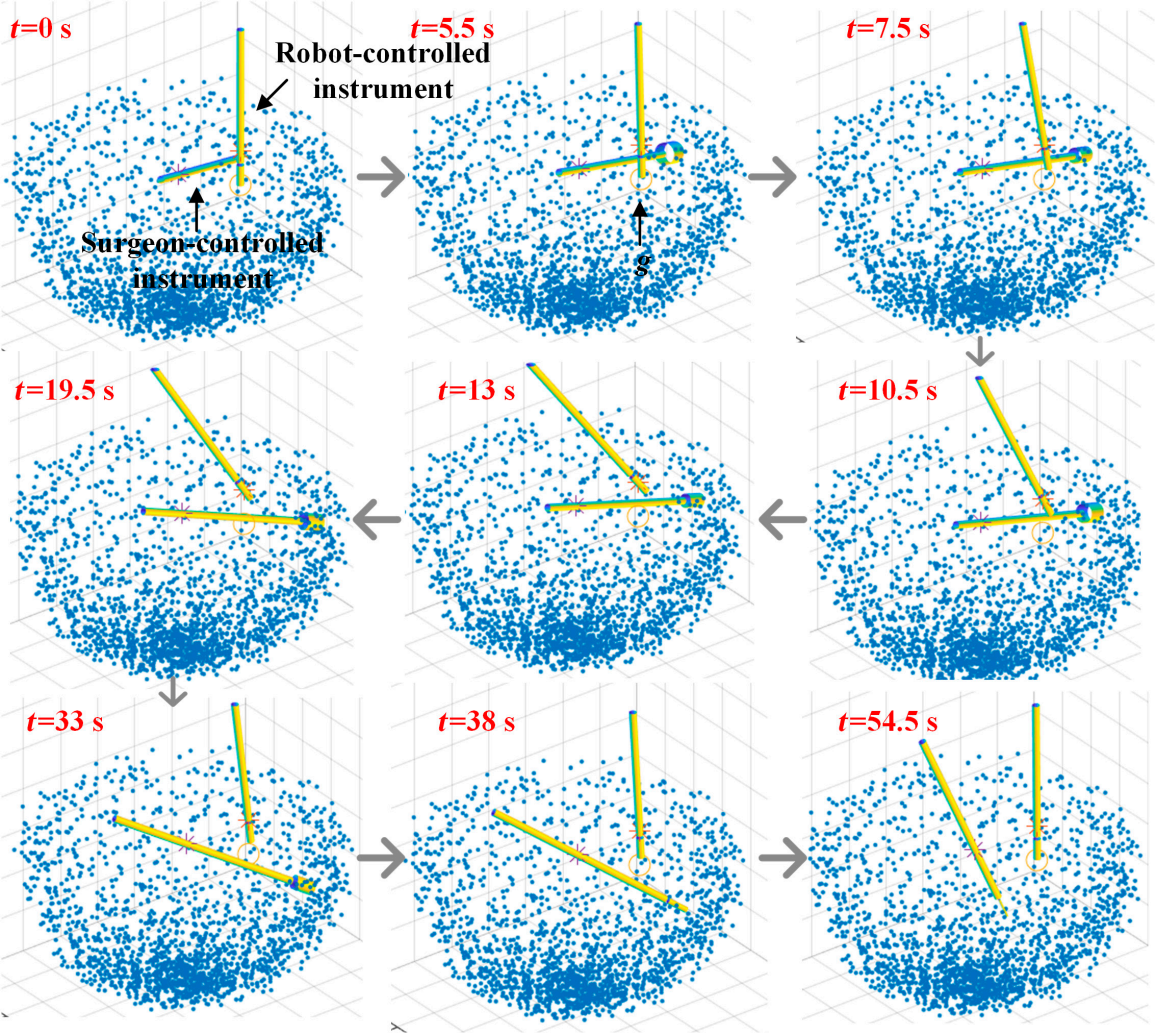
Snapshots of collision avoidance renderings.

The snapshots of collision avoidance renderings are presented in Fig. 10, where it can be seen that the proposed algorithm could drive the robot to avoid collisions automatically when the surgeon-controlled instrument was close to the robot-controlled instrument. When the robot-controlled instrument was out of danger, it continued to approach the desired position. It should be noted that the robot-controlled instrument did not touch the tissues during the performed actions.

**Fig. 11.**
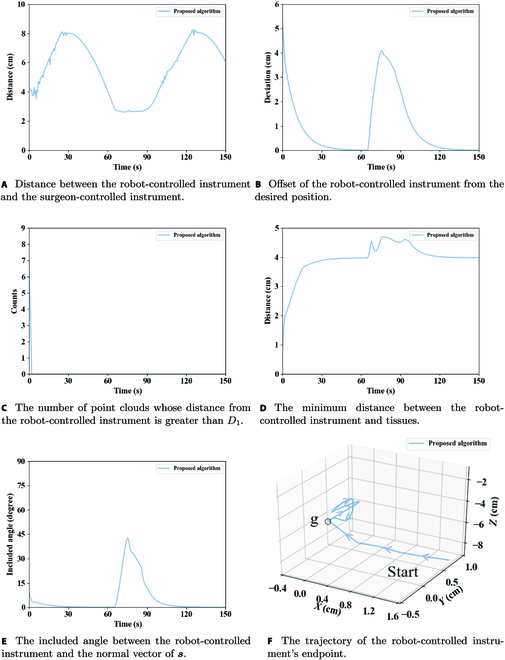
(A to F) Simulation results of the proposed algorithm.

The simulation results of the proposed algorithm are presented in Fig. 11. It can be observed in Fig. 11A that the proposed algorithm could control the robot-controlled instrument to be far away from the surgeon-controlled instrument, and the distance between the 2 instruments was not less than *D*. When the robot-controlled instrument deviated from the desired position, the desired position generated a gravitational force on the robot-controlled instrument, causing the robot-controlled instrument to approach the desired position gradually. Although in the danger-avoiding process the robot-controlled instrument might temporarily deviate from the desired position, it would return to it in a short time, as shown in Fig. 11B. Figure 11C demonstrates the number of cloud points of tissues that were close to the robot-controlled instrument, having a distance of less than *D*_1_; the minimum distance between the robot-controlled instrument and tissues is shown in Fig. 11D. The results in Fig. 11C and D indicate that the proposed algorithm could quickly move the robot-controlled instrument away from tissues. The results of the included angle between the robot-controlled instrument and the normal vector of ***s*** are presented in Fig. 11E; the result shows that the included angle was less than 45^∘^, thus ensuring collision avoidance with the upper part of tissues. The trajectory of the robot-controlled instrument’s endpoint is displayed in Fig. 11F.

### Discussion

The simulation results have shown that the proposed algorithm can not only prevent collision between surgical instruments but also prevent the robot-controlled instrument from hitting the surrounding tissues. The components of the proposed CI vector can be used individually or jointly; ***CI***_1_, ***CI***_2_, and ***CI***_3_ all can achieve collision avoidance between surgical instruments. The order of their collision avoidance effects is ***CI***_2_ > ***CI***_3_ > ***CI***_1_. The proposed CT vector can prevent the robot-controlled instrument from colliding with tissues; ***CT***_1_ shows significant performance far away from tissues, while ***CT***_2_ is mainly used to prevent a robot-controlled instrument from colliding with the upper part of tissues near the incision point.

Compared with the existing studies on collision avoidance in surgical robots, the proposed method has the following advantages:

1. The proposed algorithm considers collisions with tissues and can prevent them.
2. The proposed algorithm is suitable for both the surgeon–robot collaborative environment and for the master–slave control operating systems, while the existing collision avoidance algorithms are suitable only for the master–slave control systems.
3. Different parameter combinations can provide different collision avoidance effects, which can be adjusted adaptively according to the actual surgical environment.

The proposed method is mainly suitable for 3D collision avoidance of robot-assisted surgical systems. On the one hand, when the robot-controlled instrument is not in use, the robot-controlled instrument is prevented from colliding with other instruments and tissues so that it does not affect the surgeon’s operation. On the other hand, when the robot-controlled instrument is working, the proposed method prevents it from colliding with other instruments or tissues. Because the maximum velocity of a robot-controlled instrument is *V*max and the surgeon-controlled instrument’s velocity is uncertain, the proposed method cannot avoid all collisions. Namely, it can only minimize the possibility of collisions and reduce the damage to patients or robot-assisted surgical systems.

Due to the limitations of laboratory hardware conditions and insufficient level of in vivo 3D perception algorithms, the proposed algorithm is currently verified only by simulation experiments. In the future, the proposed algorithm will be tested on a real robotic system.

## Conclusion

This paper presents a novel 3D collision avoidance method for a robot-assisted surgical system, which can avoid collisions during a MIS. A robot-assisted surgical system is introduced, and its kinematics with the RCM constraints are analyzed. Also, an automatic collision avoidance framework is designed to prevent the in vivo collisions. In the proposed framework, the CI and CT vectors are defined to avoid collisions between surgical instruments and collision between a robot-controlled instrument and tissues. The CC vector is designed to restrict the operation of a robot-controlled instrument to perform its normal work. The proposed method can effectively avoid collisions with surgeon-controlled instruments from various directions and prevent collisions with nontarget tissues, thereby enhancing the safety of robot-assisted MIS. Finally, a collision avoidance algorithm is developed under the proposed method framework. Users can design their own algorithms as long as they adhere to the framework. The collision avoidance performance of the proposed algorithm is verified by the simulation of a MIS scene. It is analyzed how the parameters affect the collision avoidance effect and how to select optimal parameters. In addition, it is verified that the proposed algorithm can ensure collision avoidance between the robot-controlled instrument and surgeon-controlled instrument and between a robot-controlled instrument and tissues. The method and results presented in this work are beneficial to the design of robotic automatic controlling surgery instruments or an endoscope during a MIS. The proposed method can be applied to numerous medicine areas, including gynecology and urology. In the future, a real robot-assisted surgical system will be built, and the proposed method will be tested on a real system.

## Data Availability

The code and data are available at https://github.com/cynerelee/collision-avoidance.
